# Trends in COVID‐19–Attributable Hospitalizations Among Adults With Laboratory‐Confirmed SARS‐CoV‐2—COVID‐NET, June 2020 to September 2023

**DOI:** 10.1111/irv.70021

**Published:** 2024-11-04

**Authors:** Christopher A. Taylor, Michael Whitaker, Monica E. Patton, Michael Melgar, Pam Daily Kirley, Breanna Kawasaki, Kimberly Yousey‐Hindes, Kyle P. Openo, Patricia A. Ryan, Sue Kim, Kathryn Como‐Sabetti, Dominic Solhtalab, Grant Barney, Brenda L. Tesini, Nancy E. Moran, Melissa Sutton, H. Keipp Talbot, Kristen Olsen, Fiona P. Havers

**Affiliations:** ^1^ National Center for Immunization and Respiratory Diseases Centers for Disease Control and Prevention Atlanta Georgia USA; ^2^ United States Public Health Service Commissioned Corps Rockville Maryland USA; ^3^ California Emerging Infections Program Oakland California USA; ^4^ Colorado Department of Public Health & Environment Denver Colorado USA; ^5^ Connecticut Emerging Infections Program Yale School of Public Health New Haven Connecticut USA; ^6^ Emory University School of Medicine Atlanta Georgia USA; ^7^ Georgia Emerging Infections Program, Georgia Department of Public Health Atlanta Georgia USA; ^8^ Atlanta Veterans Affairs Medical Center Decatur Georgia USA; ^9^ Maryland Department of Health Baltimore Maryland USA; ^10^ Michigan Department of Health and Human Services Lansing Michigan USA; ^11^ Minnesota Department of Health St. Paul Minnesota USA; ^12^ New Mexico Emerging Infections Program Albuquerque New Mexico USA; ^13^ New York State Department of Health Albany New York USA; ^14^ University of Rochester School of Medicine and Dentistry Rochester New York USA; ^15^ Ohio Department of Health Columbus Ohio USA; ^16^ Public Health Division Oregon Health Authority Portland Oregon USA; ^17^ Vanderbilt University Medical Center Nashville Tennessee USA; ^18^ Salt Lake County Health Department Salt Lake City Utah USA

**Keywords:** COVID‐19, epidemiology, hospitalizations, population‐based surveillance

## Abstract

**Background:**

Screening for SARS‐CoV‐2 infection among hospital admissions made interpretation of COVID‐19 hospitalization data challenging as SARS‐CoV‐2–positive persons with mild or asymptomatic infection may be incorrectly identified as COVID‐19–associated hospitalizations. The study objective is to estimate the proportion of hospitalizations likely attributable to COVID‐19 among SARS‐CoV‐2–positive hospitalized patients.

**Methods:**

A sample of laboratory‐confirmed SARS‐CoV‐2–positive hospitalizations from the COVID‐19–Associated Hospitalization Surveillance Network (COVID‐NET) from June 2020 to September 2023 was analyzed, with a focus on July 2022 to September 2023. Likely COVID‐19–attributable hospitalizations were defined as hospitalizations among SARS‐CoV‐2–positive non‐pregnant adults ages ≥ 18 years with COVID‐19–related presenting complaint, treatment, or discharge diagnosis.

**Results:**

Among 44,816 sampled hospitalizations, 90% met the definition of likely COVID‐19–attributable. Among the 9866 admissions occurring during July 2022 to September 2023, 86% were likely COVID‐19–attributable; 87% had a COVID‐19–related presenting complaint, 64% received steroids or COVID‐19–related treatment, 47% had respiratory‐ and 10% had coagulopathy‐related discharge diagnoses, and 39% had COVID‐19 as the principal discharge diagnosis code. More than 70% met ≥ 2 criteria. Compared with likely COVID‐19–attributable hospitalizations, SARS‐CoV‐2–positive patients who did not meet the case definition were more likely to be ages 18–49 years (27% vs. 13%), have no underlying medical conditions (14% vs. 4%), or be asymptomatic for COVID‐19 upon admission (46% vs. 10%) (all *p* < 0.05).

**Conclusions:**

Most hospitalizations among SARS‐CoV‐2–positive adults in a recent period were likely attributable to COVID‐19. COVID‐19–attributable hospitalizations are less common among younger SARS‐CoV‐2–positive hospitalized adults but still account for nearly three quarters of all admissions among SARS‐CoV‐2–positive adults in this age group.

## 1 Introduction

After the onset of COVID‐19 pandemic in early 2020, many US hospitals implemented near‐universal screening and testing for SARS‐CoV‐2 infection among admitted patients. Because asymptomatic infection among SARS‐CoV‐2–positive persons was widespread [1], universal screening upon hospital admission identified some hospitalized patients whose admission was not directly related to their SARS‐CoV‐2 infection. Assessing the proportion of hospitalizations likely attributable to COVID‐19 among all patients admitted with a positive SARS‐CoV‐2 test is challenging, but understanding this is critical to understanding risk factors for hospitalization due to COVID‐19 as well as estimating the true burden of severe COVID‐19 disease and the ongoing impact of SARS‐CoV‐2 infections on the healthcare system.

COVID‐19 causes a broad swath of clinical outcomes, including exacerbation of chronic conditions, such as diabetes [2], chronic obstructive pulmonary disease (COPD) [3] and congestive heart failure [4], and increased hypercoagulability and thrombotic risks, including stroke [5] and myocardial infarction [4]. When assessing hospitalizations among patients with positive SARS‐CoV‐2 tests, the true burden of COVID‐19 hospitalizations is likely underestimated if hospitalizations can only be considered attributable to COVID‐19 if patients had primary respiratory presentations or received COVID‐19–specific treatments. Conversely, including hospitalizations among SARS‐CoV‐2–positive persons with no indications of COVID‐19 disease may overestimate the burden of hospitalizations attributable to COVID‐19.

Using data from a large, geographically diverse, representative public health surveillance system, we estimate the proportion of hospitalizations attributable to COVID‐19 among SARS‐CoV‐2–positive hospitalized patients and describe characteristics of those patients. Presenting complaint, discharge diagnoses, and receipt of COVID‐19–specific treatment were used to define COVID‐19–attributable hospitalization status. COVID‐19–attributable hospitalizations for July 2022 to September 2023 were described and compared with hospitalizations among SARS‐CoV‐2–positive patients whose hospitalizations were likely not attributable to COVID‐19.

## 2 Methods

COVID‐NET conducts population‐based surveillance of laboratory‐confirmed COVID‐19–associated hospitalizations among persons of all ages in > 300 acute‐care hospitals in a defined catchment area of 98 counties across 13 states (California, Colorado, Connecticut, Georgia, Maryland, Michigan, Minnesota, New Mexico, New York, Ohio, Oregon, Tennessee, and Utah) that includes approximately 10% of the US population. Hospitalizations that meet the surveillance case definition are those where the patient resides in the catchment area and receives a positive molecular or rapid antigen SARS‐CoV‐2 test result ≤ 14 days before admission or during hospitalization, either ordered by a clinician or through screening.

Using previously described methods [6], trained surveillance officers abstracted comprehensive demographic and clinical data from an age‐ and site‐stratified random sample of COVID‐19‐associated hospitalizations among patients of all ages meeting the surveillance case definition. This analysis examined hospitalizations among non‐pregnant adults ages ≥ 18 years with laboratory‐confirmed SARS‐CoV‐2 infection admitted between June 2020 and September 2023, with a more detailed analysis on the 14‐month period (July 2022 to September 2023) of data available prior to the end of the most recently concluded surveillance season that runs from October through September. Pregnant women were excluded because their standards for hospital admission differ from those for non‐pregnant persons [7]. The study period was defined by periods of SARS‐CoV‐2 variant predominance (pre‐Delta [June 1, 2020, to June 19, 2021], Delta [June 20 to December 18, 2021], early Omicron [December 19, 2021, to January 21, 2023], and later Omicron [January 22 to September 30, 2023]). Abstracted data included age, sex, race and ethnicity, long‐term care facility (LTCF) residence status, signs and symptoms on admission,^1^ underlying medical conditions, immunocompromised status, and interventions and outcomes, including intensive care unit [ICU] admission, receipt of invasive mechanical ventilation, and in‐hospital death.

### 2.1 COVID‐19–Attributable Hospitalizations

Among laboratory‐confirmed SARS‐CoV‐2–positive patients, hospitalizations likely attributable to COVID‐19 were defined as hospitalizations in which COVID‐19–related illness likely affected the course of hospitalization (Figure S1) as evidenced by either a COVID‐19–related (a) presenting complaint, (b) treatment, or (c) discharge diagnosis, as defined below. Hospitalizations likely not attributable to COVID‐19 were hospitalizations where no COVID‐19–related presenting complaint, discharge diagnosis, or treatment were found in the medical record.

#### 2.1.1 Presenting complaint

The primary presenting complaint upon admission was identified during chart abstraction using information in the admission history of present illness (HPI) or face sheet. Surveillance officers categorized patients' presenting complaints as either COVID‐19–related illness; obstetrics, including labor and delivery; planned inpatient surgery or procedures; psychiatric admission requiring acute medical care; trauma; other (with an accompanying free text field); or unknown (Figure S2). Surveillance officers categorized presenting complaints as COVID‐19–related illness if the HPI or face sheet mentioned the admission was due to fever or respiratory illness, COVID‐19–like illness, or suspicion for COVID‐19 (Table S1). “Other” presenting complaints listed in free text were reviewed and adjudicated by three physicians and recategorized as COVID‐19–related illness if they included (a) exacerbations of underlying conditions (e.g., asthma exacerbation or sickle cell crisis), (b) cardiac or neurologic complications (e.g., myocardial infarction or stroke), or (c) falls, confusion, and mental status changes. All other free text presenting complaints, including acute non‐respiratory infections that were likely independent of SARS‐CoV‐2 infection (e.g., osteomyelitis, diabetic foot abscess) and instances in which the medical chart specifically indicated that SARS‐CoV‐2 was an incidental finding or that the admission was likely not COVID‐19 related, remained categorized as “other.” Presenting complaints categorized by surveillance officers or reviewing physicians as obstetrics, labor and delivery, planned inpatient surgery/procedure, psychiatric admission, or trauma, or remained categorized as “other” were considered non‐COVID‐19–related complaints.

#### 2.1.2 Treatment

Medications recommended for the therapeutic management of hospitalized adults with COVID‐19 [8] administered during hospitalization during the study period were abstracted from the Medication Administration Report (MAR) or discharge summary within the medical record. COVID‐19 treatments were defined as in‐hospital receipt of systemic steroids (e.g., dexamethasone) or of remdesivir, baricitinib, tocilizumab, or sarilumab. Patients hospitalized for reasons other than COVID‐19 who are at high risk for progressing to severe COVID‐19 may be administered these therapies [8]. Therefore, the percent of patients who only received COVID‐19–related non‐steroidal therapy was examined separately.

#### 2.1.3 Discharge diagnoses

Two data sources within the medical chart were used to define COVID‐19–related discharge diagnoses. The first source included (*International Classification of Disease, Tenth Edition, Clinical Modification* [ICD‐10‐CM]) discharge diagnosis codes; if a COVID‐19 (code U07.1) was the principal ICD‐10‐CM discharge diagnosis code indicating COVID‐19 was “chiefly responsible for occasioning the admission of the patient,” [9] the patient was considered to have a COVID‐19–related discharge diagnosis. The second source included discharge diagnoses of specific conditions not noted upon admission that were abstracted from the discharge summary and not reliant on administrative medical billing codes. COVID‐19–related discharge diagnoses from the discharge summary were defined as (a) respiratory‐related discharge diagnoses (acute respiratory distress syndrome [ARDS], acute respiratory failure, asthma exacerbation, bronchiolitis, bronchitis, chronic obstructive pulmonary disease [COPD] exacerbation, pneumonia, or sepsis) and (b) coagulopathy‐related discharge diagnoses (acute myocardial infarction, deep vein thrombosis, disseminated intravascular coagulation [DIC], pulmonary embolism, stroke/cerebrovascular accident, or other thrombosis, embolism, or coagulopathy).

Unweighted case counts are presented with weighted percentages unless otherwise noted to better represent the hospitalized population of the catchment area by accounting for sampling and nonresponse (defined as an incomplete chart review). Weighted case counts of hospitalizations are calculated to estimate the number of hospitalizations attributable to COVID‐19 by age group and month. Weighted case counts are calculated by applying weighted point estimates to unweighted counts to demonstrate the estimated magnitude of total hospitalizations by age groups within variant periods. Data were analyzed using SAS (version 9.4; SAS Institute); variances were estimated using Taylor series linearization method. Statistical differences between groups were assessed using chi‐square tests; *p* < 0.05 was considered statistically significant. This activity was reviewed by CDC and conducted consistent with applicable federal law and CDC policy.^2^

## 3 Results

From June 2020 to September 2023, 434,884 hospitalizations among SARS‐CoV‐2–positive adults met the criteria for inclusion in COVID‐NET. Of those, a sample of 49,079 (11.3%) hospitalizations underwent complete medical chart abstraction. After excluding patients identified as pregnant or presenting with a complaint related to obstetrics or labor and delivery (4263 [8.7%]), 44,816 total sampled hospitalizations were included in this analysis. Among these, 90.2% were likely attributable to COVID‐19 across non‐mutually exclusive categories, including 36,822 (84.2%) with COVID‐19–related presenting complaints, 29,288 (67.5%) with COVID‐19–related treatments, and 31,681 (72.6%) with COVID‐19–related discharge diagnoses. Among likely COVID‐19–attributable hospitalizations, 6826 (14.9%), 8941 (19.4%), and 24,361 (55.9%) met one, two, or three of these criteria, respectively.

Among 9866 SARS‐CoV‐2–positive adults hospitalized between July 2022 and September 2023, 14.9% were ages 18–49 years and 66.0% were ages ≥ 65 years (Table 1). Most were non‐Hispanic White (63.0%); 19.8% identified as non‐Hispanic Black and 8.0% as Hispanic persons. More than half (63.9%) had ≥ 3 underlying medical conditions; 15.4% resided in an LTCF at admission. Of these 9866 hospitalizations, 8351 (86.2%) met criteria as likely COVID‐19–attributable hospitalizations; 76.6% met criteria with a COVID‐19–related presenting complaint, 55.8% with COVID‐19–specific inpatient treatment or systemic steroids, and 60.7% with a COVID‐19–related discharge diagnosis, including 40.0% and 8.2% who received a pulmonary or coagulopathy‐related discharge diagnosis per the discharge summary, respectively, and 33.9% with COVID‐19 as the principal discharge ICD‐10‐CM code.

**TABLE 1:** Characteristics of hospitalizations with laboratory‐confirmed positive SARS‐CoV‐2 tests performed ≤ 14 days before or during hospitalization among non‐pregnant adults ages ≥ 18 years overall and by COVID‐19–attributable hospitalization status—COVID‐NET, 13 States, July 2022 to September 2023.

Characteristic	Overall	Hospitalizations likely attributable to COVID‐19^a^	Hospitalizations that are likely not COVID‐19–attributable^b^	*p* value
*N*	9866 (100%)	8351, 86.2% (85.0–87.4)	1515, 13.8% (12.6–15.0)	
	*n*, % (95% CI)	*n*, % (95% CI)	*n*, % (95% CI)	
Age group				< 0.0001
18–49 years	1984, 14.9% (13.9–16.0)	1470, 13.0% (11.9–14.1)	514, 27.3% (23.9–30.9)	
50–64 years	3196, 19.0% (18.0–20.1)	2700, 18.4% (17.3–19.6)	496, 22.8% (19.7–26.0)	
65–74 years	1961, 21.7% (20.3–23.1)	1726, 21.9% (20.4–23.5)	235, 20.4% (16.8–24.4)	
≥ 75 years	2725, 44.3% (42.5–46.2)	2455, 46.7% (44.7–48.7)	270, 29.6% (24.8–34.7)	
Sex				0.7049
Male	4968, 49.9% (48.1–51.7)	4166, 49.8% (47.8–51.7)	802, 50.7% (46.1–55.3)	
Female	4898, 50.1% (48.3–51.9)	4185, 50.2% (48.3–52.2)	713, 49.3% (44.7–53.9)	
Race and Ethnicity				0.0632
White, non‐Hispanic	6632, 63.0% (61.3–64.8)	5671, 63.7% (61.8–65.6)	961, 58.8% (54.2–63.2)	
Black, non‐Hispanic	1703, 19.8% (18.4–21.3)	1425, 19.4% (17.9–21.1)	278, 22.4% (18.6–26.6)	
American Indian/Alaska Native, non‐Hispanic	157, 0.9% (0.7–1.2)	119, 0.9% (0.7–1.2)	38, 1.1% (0.6–1.8)	
Asian, Native Hawaiian, or other Pacific Islander, non‐Hispanic	334, 5.0% (4.1–6.1)	293, 5.2% (4.2–6.4)	41, 3.8% (2.2–6.2)	
Hispanic, any race	792, 8.0% (7.1–9.0)	655, 7.8% (6.8–8.8)	137, 9.4% (7.3–12.0)	
Other or unknown race, non‐Hispanic	248, 3.1% (2.5–3.9)	188, 2.9% (2.3–3.7)	60, 4.4% (2.8–6.6)	
Resident of a long‐term care facility	1237, 15.4% (14.0–16.8)	1115, 16.0% (14.6–17.6)	122, 10.5% (7.7–14.0)	0.0049
Number of underlying medical conditions				< 0.0001
0	715, 5.6% (5.0–6.2)	485, 4.3% (3.8–5.0)	230, 14.4% (11.8–17.4)	
1	1286, 11.3% (10.3–12.4)	1056, 11.0% (9.8–12.2)	230, 13.9% (11.1–17.1)	
2	1813, 19.2% (17.8–20.7)	1572, 19.2% (17.7–20.7)	241, 19.6% (15.2–24.5)	
≥ 3	5782, 63.9% (62.2–65.6)	5213, 65.6% (63.7–67.3)	569, 52.1% (47.1–57.1)	
Immunocompromised status	1685, 16.1% (14.9–17.3)	1554, 16.8% (15.5–18.1)	131, 11.3% (8.3–15.0)	0.0069
Interventions and outcomes for severe disease				
Intensive care unit admission	1650, 15.2% (14.0–16.5)	1507, 15.8% (14.5–17.1)	143, 11.3% (8.3–14.8)	0.0207
Invasive mechanical ventilation	672, 6.1% (5.4–7.0)	623, 6.5% (5.7–7.4)	49, 3.5% (2.2–5.2)	0.0026
In‐hospital death	479, 4.1% (3.4–4.7)	399, 4.3% (3.6–5.1)	80, 2.4% (1.5–3.6)	0.0083
Presenting complaint upon admission^c^				—
COVID‐19–related illness	7181, 76.6% (75.1–78.1)	7181, 87.4% (86.1–88.5)	0, 0%^d^	
Inpatient surgery or procedure	263, 2.5% (2.0–3.2)	133, 1.6% (1.1–2.2)	130, 9.5% (7.1–12.3)	
Psychiatric admission requiring acute medical care	364, 2.4% (2.0–2.8)	98, 0.9% (0.7–1.2)	266, 12.8% (10.5–15.3)	
Trauma	247, 2.2% (1.6–2.9)	126, 0.9% (0.7–1.2)	121, 11.0% (7.3–15.8)	
Other	1539, 16.3% (15.0–17.6)	787, 9.2% (8.2–10.3)	752, 66.7% (61.9–71.3)	
COVID‐19–associated signs and symptoms				
≥ 1 respiratory sign or symptom^e^	5763, 58.5% (56.7–60.2)	5525, 65.4% (63.5–67.2)	238, 15.2% (12.1–18.7)	< 0.0001
≥ 1 non‐respiratory sign or symptom^f^	7280, 75.5% (73.9–76.9)	6589, 79.4% (77.7–80.9)	691, 51.0% (46.5–55.6)	< 0.0001
≥ 1 respiratory^e^ or non‐respiratory sign or symptom^f^	8262, 85.2% (84.0–86.3)	7521, 90.2% (89.1–91.3)	741, 53.6% (49.1–58.1)	< 0.0001
No respiratory^e^ or non‐respiratory^f^ sign or symptom (asymptomatic)	1604, 14.8% (13.7–16.0)	830, 9.8% (8.7–10.9)	774, 46.4% (41.9–50.9)	< 0.0001
COVID‐19 treatment				
COVID‐19–specific inpatient treatment^g^	3149, 36.1% (34.4–37.9)	3149, 41.2% (39.3–43.2)	0, 0%^d^	—
Systemic steroids	4381, 43.1% (41.4–44.9)	4381, 49.2% (47.3–51.1)	0, 0%^d^	—
Either COVID‐19–specific treatment^g^ or systemic steroids	5299, 55.8% (54.0–57.6)	5299, 63.7% (61.8–65.5)	0, 0%^d^	—
COVID‐19–related discharge diagnosis				
Pulmonary discharge diagnosis^h^	3521, 40.0% (38.3–41.8)	3521, 46.4% (44.5–48.3)	0, 0%^d^	—
Coagulopathy‐related discharge diagnosis^i^	679, 8.2% (7.2–9.2)	679, 9.5% (8.4–10.6)	0, 0%^d^	—
COVID‐19 (U07.1) as principal ICD‐10‐CM discharge code	2999, 33.9% (32.2–35.6)	2999, 39.3% (37.4–41.2)	0, 0%^d^	—
Any COVID‐19–related discharge diagnosis	5503, 60.7% (59.0–62.4)	5503, 70.4% (68.7–72.1)	0, 0%^d^	—

*Note:* All counts presented are unweighted; all percentages are weighted.

Abbreviations: COVID‐19, Coronavirus Disease‐2019; COVID‐NET, COVID‐19–Associated Hospitalization Surveillance Network; ICD‐10‐CM, International Classification of Diseases, Tenth Edition, Clinical Modification; SARS‐CoV‐2, severe acute respiratory syndrome coronavirus 2.

^a^ Likely COVID‐19–attributable hospitalizations are defined as hospitalizations among laboratory‐confirmed SARS‐CoV‐2–positive patients with the test administered ≤ 14 days before or during admission with COVID‐19–related presenting complaint, treatment, or discharge diagnosis.

^b^ Hospitalizations likely not attributable to COVID‐19 have no COVID‐19–related presenting complaint, treatment, or discharge diagnosis.

^c^ Presenting complaint upon admission was identified using information in the admission history and physical or face sheet. Presenting complaints are categorized as COVID‐19–related illness; obstetrics, including labor and delivery; inpatient surgery or procedures; psychiatric admission requiring acute medical care; trauma; other (with an accompanying free‐text field); or unknown. Five clinicians independently reviewed the free‐text field of complaints classified as other to determine if the complaint should be recategorized or remain in the “other” category (e.g., skin and soft tissue infections) (Table S1). In general, exacerbations of underlying conditions (e.g., asthma exacerbation or sickle cell crisis) and cardiac or neurologic complications (e.g., myocardial infarction or stroke) were classified as COVID‐19–related. For adults ages ≥ 50 years, falls, confusion, and mental status changes were classified as COVID‐19–related. For classifications not in agreement across all reviewers, adjudication was done to reach consensus. Hospitalizations with unknown presenting complaint upon admission represent < 1% of hospitalizations.

^d^ By definition.

^e^ COVID‐19–related respiratory signs and symptoms included congestion, cough, hemoptysis, shortness of breath, sore throat, upper respiratory infection, influenza‐like illness, and wheezing.

^f^ COVID‐19–related non‐respiratory signs and symptoms of COVID‐19 included abdominal pain, altered mental status, anosmia, chest pain, conjunctivitis, diarrhea, dysgeusia taste, fatigue, fever/chills, headache, myalgia, nausea/vomiting, rash, and seizures.

^g^ Includes remdesivir, baricitinib, sarilumab, and tocilizumab.

^h^ Pulmonary discharge diagnoses were defined as acute respiratory distress syndrome (ARDS), acute respiratory failure, asthma exacerbation, bronchiolitis, bronchitis, chronic obstructive pulmonary disease (COPD) exacerbation, pneumonia, or sepsis.

^i^ Coagulopathy‐related discharge diagnoses were defined as acute myocardial infarction, deep vein thrombosis, disseminated intravascular coagulation (DIC), pulmonary embolism, stroke/cerebrovascular accident, or other thrombosis, embolism, or coagulopathy.

Among the 8351 likely COVID‐19–attributable hospitalizations, 87.4% met criteria with a COVID‐19–related presenting complaint, 63.7% with COVID‐19–specific inpatient treatment or systemic steroids, and 65.9% with a COVID‐19–related discharge diagnosis, including 46.4% and 9.5% with a pulmonary or coagulopathy‐related discharge diagnosis, respectively, and 39.3% with COVID‐19 as the principal ICD‐10‐CM discharge diagnosis code. Among these 8351 likely COVID‐19–attributable hospitalizations, 2318 (27.8%), 2434 (29.1%), and 3599 (43.1%) met one, two, and three of these criteria, respectively. Among those who received COVID‐19–related treatment, not including systemic steroids, 97.1% met ≥ 1 other criterion for likely COVID‐19–attributable hospitalization (including receipt of systemic steroids); 2.9% met no other criteria.

Among 7181 hospitalizations with a COVID‐19–related presenting complaint, 63.0% and 71.6% also met criteria with either COVID‐19–related treatment or a COVID‐19–related discharge diagnosis, respectively (Table 2). Among 2413 hospitalizations with non‐COVID‐19–related presenting complaints, more than one third in each category met the criteria as a likely COVID‐19–attributable hospitalization, including 53.9% of admissions for inpatient surgery/procedure, 33.6% for trauma, 37.9% for psychiatric admissions, and 49.6% of admissions other or unknown categories of presenting complaint. Among hospitalizations that met criteria with COVID‐19–specific inpatient treatment or systemic steroids, 86.4% also had a COVID‐19–related presenting complaint. Additionally, 89.7% of hospitalizations that met criteria with a pulmonary discharge and 83.9% with a coagulopathy‐related discharge diagnosis also had a COVID‐19–related presenting complaint.

**TABLE 2:** Characteristics of hospitalizations with laboratory‐confirmed positive SARS‐CoV‐2 tests performed ≤ 14 days before or during hospitalization among non‐pregnant adults ages ≥ 18 years included in subcategories of hospitalization type based on presenting complaint and other clinical factors—COVID‐NET^a^, July 2022 to September 2023.

Characteristic	Unweighted count	Hospitalizations likely attributable to COVID‐19^b^	Hospitalizations with a COVID‐19–related presenting complaint^c^	Hospitalizations with COVID‐19–related treatment^d^	Hospitalizations with COVID‐19–related discharge diagnoses^e^
*N*	9866	8351, 86.2% (85.0–87.4)	7181, 76.6% (75.1–78.1)	5299, 55.8% (54.0–57.6)	5503, 60.7% (59.0–62.4)
		*n*, % (95% CI)	*n*, % (95% CI)	*n*, % (95% CI)	*n*, % (95% CI)
Presenting complaint upon admission^c^					
COVID‐19–related illness	7181	7181, 100.0% (100.0–100.0)	7181, 100.0% (100.0–100.0)	4495, 63.0% (61.0–65.0)	4812, 71.6% (69.8–73.4)
Inpatient surgery or procedure	263	133, 53.9% (42.5–64.9)	0, 0%^f^	122, 46.2% (34.8–57.9)	29, 12.4% (5.1–24.0)^g^
Psychiatric admission requiring acute medical care	364	98, 33.6% (26.0–41.8)	0, 0%^f^	46, 13.4% (8.4–19.9)	73, 27.6% (20.3–35.9)
Trauma	247	126, 37.9% (26.7–50.2)	0, 0%^f^	101, 29.2% (19.8–40.1)	51, 17.6% (11.0–26.0)
Other	1539	787, 49.6% (45.3–53.8)	0, 0%^f^	531, 33.4% (29.5–37.5)	513, 33.6% (29.7–37.7)
COVID‐19 treatment					
COVID‐19–specific inpatient treatment^h^	3149	3149, 100.0% (100.0–100.0)	2765, 88.9% (86.9–90.7)	3149, 100.0% (100.0–100.0)	2453, 81.8% (79.3–84.2)
Systemic steroids	4381	4381, 100.0% (100.0–100.0)	3758, 87.3% (85.5–88.9)	4381, 100.0% (100.0–100.0)	3373, 81.3% (79.2–83.3)
Either COVID‐19–specific inpatient treatment^h^ or systemic steroids	5299	5299, 100.0% (100.0–100.0)	4495, 86.4% (84.8–88.0)	5299, 100.0% (100.0–100.0)	3924, 78.4% (76.3–80.4)
COVID‐19–related discharge diagnosis^e^					
Pulmonary discharge diagnosis	3521	3521, 100.0% (100.0–100.0)	3107, 89.7% (88.1–91.2)	2849, 80.8% (78.4–83.0)	3521, 100.0% (100.0–100.0)
Coagulopathy‐related discharge diagnosis	679	679, 100.0% (100.0–100.0)	551, 83.9% (79.0–88.0)	382, 52.9% (46.7–59.0)	679, 100.0% (100.0–100.0)
COVID‐19 (U07.1) as first‐listed ICD‐10‐CM discharge code	2999	2999, 100.0% (100.0–100.0)	2753, 93.4% (92.0–94.7)	2180, 73.7% (70.7–76.4)	2999, 100.0% (100.0–100.0)
Any COVID‐19–related discharge diagnosis ^i^	5503	5503, 100.0% (100.0–100.0)	4812, 88.9% (87.5–90.2)	3924, 70.9% (68.7–73.0)	5503, 100.0% (100.0–100.0)
Hospitalizations with a COVID‐19–related presenting complaint^c^	7181	7181, 100.0% (100.0–100.0)	7181, 100.0% (100.0–100.0)	4495, 63.0% (61.0–65.0)	4812, 71.6% (69.8–73.4)
Hospitalizations with COVID‐19–related treatment^d^ or discharge diagnoses^e^	6878	6878, 100.0% (100.0–100.0)	5708, 85.0% (83.5–86.4)	5299, 75.7% (73.78–77.5)	5503, 82.7% (82.1–85.2)
COVID‐19–attributable hospitalizations^b^	8351	8351, 100% (100.0–100.0)	7181, 87.4% (86.1–88.5)	5299, 63.7% (61.8–65.5)	5503, 70.4% (68.7–72.1)

*Note:* All counts presented are unweighted; all percentages are weighted.

Abbreviations: COVID‐19, coronavirus disease‐2019; COVID‐NET, COVID‐19–Associated Hospitalization Surveillance Network; ICD‐10‐CM, International Classification of Diseases, Tenth Edition, Clinical Modification; SARS‐CoV‐2, severe acute respiratory syndrome coronavirus 2.

^a^ Selected counties in California, Colorado, Connecticut, Georgia, Maryland, Michigan, Minnesota, New Mexico, New York, Ohio, Oregon, Tennessee, and Utah.

^b^ COVID‐19–attributable hospitalizations are defined as hospitalizations among patients with a positive laboratory‐confirmed SARS‐CoV‐2 test administered ≤14 days before or during admission where COVID‐19–related illness (a) could be plausibly associated with the presenting complaint or likely affected the course of hospitalization. Hospitalizations where COVID‐19–related illness likely affected the clinical course were defined as including (a) pulmonary discharge diagnoses (defined as acute respiratory distress syndrome [ARDS], acute respiratory failure, asthma exacerbation, bronchiolitis, bronchitis, chronic obstructive pulmonary disease [COPD] exacerbation, pneumonia, or sepsis); (b) coagulopathy‐related discharge diagnoses (acute myocardial infarction, deep vein thrombosis, disseminated intravascular coagulation [DIC], pulmonary embolism, stroke/cerebrovascular accident, or other thrombosis, embolism, or coagulopathy); (c) COVID‐19 (code U07.1) listed as the first‐listed ICD‐10‐CM discharge diagnosis code; (d) in‐hospital receipt of systemic steroids; or (e) receipt of medications recommended for the therapeutic management of hospitalized adults with COVID‐19.

^c^ Presenting complaint upon admission was identified using information in the admission history and physical or face sheet. Presenting complaints are categorized as COVID‐19–related illness; obstetrics, including labor and delivery; inpatient surgery or procedures; psychiatric admission requiring acute medical care; trauma; other (with an accompanying free‐text field); or unknown. Five clinicians independently reviewed the free‐text field of complaints classified as other to determine if the complaint should be recategorized or remain in the “other” category (e.g., skin and soft tissue infections) (Table S1). In general, exacerbations of underlying conditions (e.g., asthma exacerbation or sickle cell crisis) and cardiac or neurologic complications (e.g., myocardial infarction or stroke) were classified as COVID‐19–related. For adults ages ≥ 50 years, falls, confusion, and mental status changes were classified as COVID‐19–related. For classifications not in agreement across all reviewers, adjudication was done to reach consensus. Hospitalizations with unknown presenting complaint upon admission represent < 1% of hospitalizations.

^d^ Defined as receipt of remdesivir, baricitnib, sarilumab, tocilizumab, or systemic steroids.

^e^ Includes pulmonary discharge diagnoses, defined as acute respiratory distress syndrome (ARDS), acute respiratory failure, asthma exacerbation, bronchiolitis, bronchitis, chronic obstructive pulmonary disease (COPD) exacerbation, pneumonia, or sepsis, or coagulopathy‐related discharge diagnoses, defined as acute myocardial infarction, deep vein thrombosis, disseminated intravascular coagulation (DIC), pulmonary embolism, stroke/cerebrovascular accident, or other thrombosis, embolism, or coagulopathy.

^f^ By definition.

^g^ Defined as receipt of remdesivir, baricitnib, sarilumab, or tocilizumab.

^h^ Includes pulmonary or coagulopathy‐related discharge diagnoses or COVID‐19 (U07.1) as first‐listed ICD‐10‐CM discharge code.

^i^ Relative standard error > 30%; estimate might be unstable due to limited sample size.

The remaining 1515 hospitalizations (13.8%) lacked a COVID‐19–related presenting complaint, treatment, and discharge diagnosis and were likely not attributable to COVID‐19. Among these, 9.5%, 12.8%, and 11.0% of presenting complaints were related to inpatient surgery/procedure, psychiatric admission, and trauma, respectively; the remaining (66.7%) were classified as other. Compared with likely COVID‐19–attributable hospitalizations, hospitalizations likely not attributable to COVID‐19 were more common among adults ages 18–49 years than among those ages ≥ 50 years (27.3% vs. 13.0%), among patients with no underlying medical conditions (14.4% vs. 4.3%), and among patients who presented with no COVID‐19‐associated signs or symptoms at admission (46.4% vs. 9.8%) (all *p* < 0.0001). Compared with hospitalizations likely not attributable to COVID‐19, hospitalizations likely attributable to COVID‐19 were more likely to occur among:adults ages ≥ 75 years (46.7% vs. 29.6%, p < 0.0001), LTCF residents (16.0% vs. 10.5%, *p* = 0.0049), persons with immunocompromising conditions (16.8% vs. 11.3%, *p* = 0.0069), and patients admitted to the ICU (15.8% vs. 11.3%, *p* = 0.021), who received invasive mechanical ventilation (6.5% vs. 3.5%, *p* = 0.0026), and who died in‐hospital (4.3% vs. 2.4%, *p* = 0.0083) (Table 1).

**FIGURE 1:**
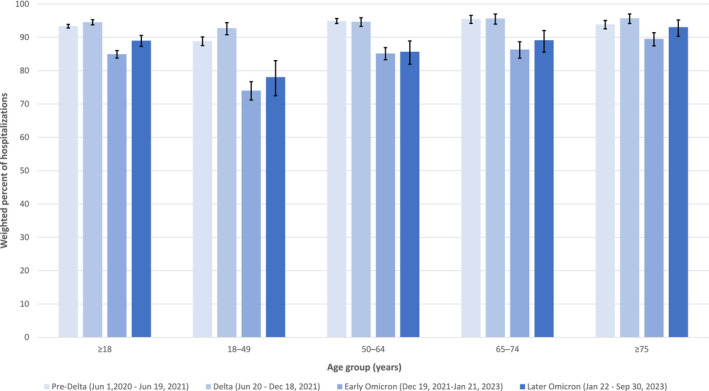
Percentage of hospitalizations likely attributable to COVID‐19 among adults ages ≥ 18 years with laboratory‐confirmed SARS‐CoV‐2, by age group and period of SARS‐CoV‐2 variant predominance—COVID‐19–Associated Hospitalization Surveillance Network (COVID‐NET), June 2020 to September 2023. Percentages are weighted to account for sampling with 95% confidence intervals indicated by error bars. Likely COVID‐19–attributable hospitalizations are defined as hospitalizations among laboratory‐confirmed SARS‐CoV‐2–positive patients with the test administered ≤ 14 days before or during admission with COVID‐19–related presenting complaint, treatment, or discharge diagnosis.

The proportion of likely COVID‐19–attributable hospitalizations among those with laboratory‐confirmed SARS‐CoV‐2 changed over the course of the pandemic. Among all sampled hospitalizations, 20,222 (93.4% [95% CI 92.8%–93.9%]) and 6338 (94.5% [95% CI 93.7%–95.3%]) of adults had a likely COVID‐19–attributable hospitalization during the pre‐Delta and Delta variant predominance periods, respectively (Figure 1). In the early and later Omicron variant periods, the proportion of COVID‐19–attributable hospitalizations among SARS‐CoV‐2–positive adults was 85.2% (95% CI 83.5%–86.7%) and 86.2% (95% CI 85.0%–87.4%), respectively. Across all adult age groups, the proportion of all COVID‐NET hospitalizations attributable to COVID‐19 was lowest during the early Omicron period of December 2021 to January 2023 and then increased in the subsequent later Omicron period (January to September 2023), but that change was only significant for adults ages 50–64 years (*p* = 0.02, *p* > 0.05 for all others).

The changes in the proportion of adults with likely COVID‐19–attributable hospitalizations over the course of the pandemic varied by age group. Adults ages ≥ 75 years had the highest proportion of hospitalizations attributable to COVID‐19, with > 89% during each variant period. Compared with adults ages > 50 years, adults ages 18–49 years had a lower proportion of total COVID‐19–attributable hospitalizations for all periods of SARS‐CoV‐2 variant predominance. Similarly, adults ages 18–49 years had lower overall proportions of hospitalizations with COVID‐19–related presenting complaints and either receipt of COVID‐19–related treatment or COVID‐19–related discharge diagnosis compared with older age groups (Figure S3). Among adults ages 18–49 years with a hospitalization likely not attributable to COVID‐19 in the later Omicron period, the most common presenting complaint was “other” (*n* = 585, 45.2% [95% CI 39.8%–50.5%]) followed by psychiatric admissions requiring acute medical care (*n* = 538, 31.6% [95% CI 27.7%–35.5%]), trauma (*n* = 252, 12.2% [95% CI 9.4%–15.0%]), and planned inpatient surgery/procedures (*n* = 198, 11.0% [95% CI 8.6%–13.5%]).

**FIGURE 2:**
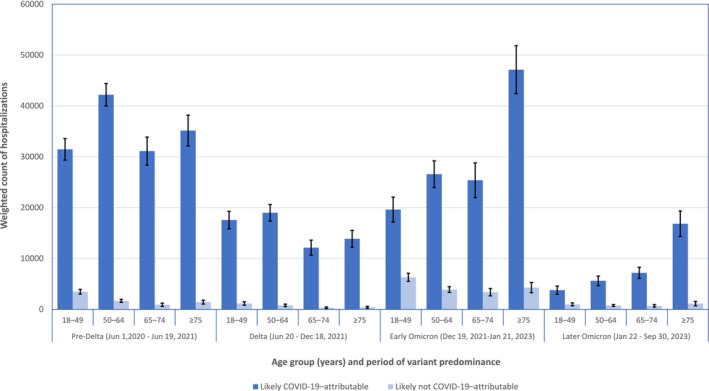
Counts of hospitalizations likely attributable and not attributable to COVID‐19 among adults ages ≥ 18 years with laboratory‐confirmed SARS‐CoV‐2, by age group and period of SARS‐CoV‐2 variant predominance—COVID‐19–Associated Hospitalization Surveillance Network (COVID‐NET), June 2020 to September 2023. Counts are weighted to account for sampling with 95% confidence intervals indicated by error bars. Likely COVID‐19–attributable hospitalizations are defined as hospitalizations among laboratory‐confirmed SARS‐CoV‐2–positive patients with the test administered ≤ 14 days before or during admission with COVID‐19–related presenting complaint, treatment, or discharge diagnosis. Hospitalizations likely not attributable to COVID‐19 have no COVID‐19‐related presenting complaint, treatment, or discharge diagnosis.

While the proportion of hospitalizations likely not attributable to COVID‐19 increased over time among adults ages 18–49 years, this age group comprised an increasingly smaller proportion of all hospitalizations among SARS‐CoV‐2–positive persons (Figure 2), decreasing from 23.7% (unweighted) of all hospitalizations in the pre‐Delta period to 13.0% (unweighted) in the later Omicron period. Hospitalizations likely not attributable to COVID‐19 in this age group accounted for 2.9% of all hospitalizations among SARS‐CoV‐2–positive patients in the later Omicron period. Conversely, the proportion of COVID‐19–attributable hospitalizations among older adults—the group comprising most hospitalizations—remained high throughout the pandemic as COVID‐19–attributable hospitalizations among adults ages ≥ 65 and ≥ 75 years accounted for 64.0% and 44.8% (unweighted) of all SARS‐CoV‐2–positive hospitalizations among non‐pregnant adults during the later Omicron period (increased from 46.5% and 24.8% in the pre‐Delta period), respectively.

## 4 Discussion

In a representative sample of > 49,000 cases from active multistate population‐based surveillance of SARS‐CoV‐2–positive hospitalizations among non‐pregnant adults, since June 2020, most hospitalizations (90%) among SARS‐CoV‐2–positive adults met the case definition as likely COVID‐19–attributable. While this proportion decreased somewhat over time, especially in younger adults aged 18–49 years, the proportion with COVID–attributable hospitalizations remained high overall (86.4%) in July 2022 to September 2023. This was likely driven by the shift in hospitalizations towards older age groups; those ages ≥ 65 years had the highest proportion of COVID‐19–attributable hospitalizations of any age group and also accounted for nearly two‐thirds of all SARS‐CoV‐2–positive hospitalizations in that period. Even among SARS‐CoV‐2–positive patients who initially presented with non‐COVID‐19–related complaints such as trauma or surgery, more than a third of each category had their course complicated by COVID‐19. This was demonstrated by receipt of COVID‐19–related inpatient treatment or a COVID‐19–related discharge diagnosis such as pneumonia or respiratory failure, underscoring the importance of multiple types of data to assess the role of COVID‐19 in hospitalizations. Hospitalizations among SARS‐CoV‐2–positive adults that were not likely attributable to COVID‐19 were more common among adults ages 18–49 years, those with no underlying medical conditions, and those presenting without COVID‐19–related symptoms upon admission.

The proportion of hospitalizations likely COVID‐19–attributable among all SARS‐CoV‐2–positive hospitalizations found in this analysis is higher than others previously reported. Using administration of a systemic steroid (dexamethasone) as a marker of severe disease, it was reported in the press in January 2022 that one US jurisdiction defined nearly half (47.8%) of hospitalizations among SARS‐CoV‐2–positive patients as incidental (indicating a patient was hospitalized for non‐COVID reasons) rather than primary [10]. A similar proportion of hospitalizations among SARS‐CoV‐2–positive adults in this analysis received systemic steroids. However, using systemic steroid administration as the sole indicator of COVID‐19–attributable hospitalization would have excluded one third of hospitalizations defined as likely COVID‐19–attributable in this analysis, as only 64.1% received such treatment in this study sample. Another study used a sample of manually reviewed electronic health records from March 2020 to August 2021 and found approximately 26% of SARS‐CoV‐2 PCR–positive hospitalizations were classified as “incidental” using an algorithm looking at admission notes, discharge summary, and lab values [11]. The wider definition of treatments, including systemic steroids, and additional COVID‐19–related discharge diagnoses used in the current study might explain the higher proportion found in this analysis. Another published study using data from April 2020 to June 2022 that used extraction from an electronic health records database and focused on ICD‐10‐CM codes and in‐hospital treatment, found 84% of pre‐Omicron and 63% of Omicron‐era hospitalizations in SARS‐CoV‐2–positive patients of all ages were primarily due to COVID‐19 [12]. These are smaller proportions than what was found in this study, even when we limit the results to those with just COVID‐19–related treatments or outcomes; this might result from the lack of reason for admission, inclusion of fewer coagulopathy‐related diagnoses, and the inclusion of children and adolescents. Another study examining a sample of hospitalization records from 2022 occurring in three German tertiary care hospitals reported that a team of providers using clinical judgment to review medical records identified 40% of SARS‐CoV‐2–positive hospitalizations as due to COVID‐19. Statistical models developed as part of this study, incorporating admission diagnosis code, admission ward, receipt of oxygen therapy, viral load, and select laboratory values, provided good sensitivity (88%) and specificity (81%) in identifying hospitalizations due to COVID‐19 [13]. Not all the factors identified in that study are available to examine in this analysis, so a direct comparison to those results cannot be provided. However, US hospital admission, diagnosis coding, and treatment practices might also contribute to differences in these results relative to similar studies performed in other countries. Further, using the wider definition of COVID‐19–attributable hospitalizations, including reason for admission and discharge diagnoses in addition to COVID‐19–related treatment likely contributed to larger proportions of COVID‐19–attributable hospitalizations found in this analysis relative to similar studies. The goal of this analysis was to define hospitalizations attributable to COVID‐19–related illness. By defining hospitalizations where COVID‐19 likely affected the course of hospitalization, including conditions known to be exacerbated by COVID‐19, this approach might be broader than other analyses where primary cases are defined as those where COVID‐19 was the sole reason for admission or otherwise played a secondary role to another medical issue. While the definition developed for this study might be broader than other approaches, more than 70% of patients still met at least two of the criteria for a COVID‐19 as a likely contributor to hospitalization.

The proportion of COVID‐19–attributable hospitalizations varied by age group, with more hospitalizations likely not attributable to COVID‐19 among younger adults. While the proportion of hospitalizations likely not attributable to COVID‐19 among hospitalized SARS‐CoV‐2–positive adults ages 18–49 years increased over time, this age group accounted for a small overall proportion of SARS‐CoV‐2‐postive hospitalizations. Thus, hospitalizations not attributable to COVID‐19 among adults ages 18–49 accounted for < 3% of all SARS‐CoV‐2–positive hospitalizations in the later Omicron period. Seen here, as well as previously reported [14], nearly two‐thirds of all SARS‐CoV‐2–positive hospitalizations in the later Omicron period are among adults ≥ 65 years, and more than 85% of hospitalizations among SARS‐CoV‐2–positive adults ages ≥ 65 years in this analysis are likely COVID‐19–attributable. As adults ages ≥ 65 years now comprise nearly two‐thirds of hospitalizations among SARS‐CoV‐2–positive adults, and most of those hospitalizations are attributable to COVID‐19, the proportion of total SARS‐CoV‐2–positive hospitalizations attributable to COVID‐19 will likely remain high absent dramatic changes in testing practices or in the epidemiology of hospitalizations among SARS‐CoV‐2–positive persons. This analysis focused solely on non‐pregnant adults; trends in COVID‐19–attributable hospitalizations among infants, children, adolescents, and pregnant adults differ from those presented in this analysis; other COVID‐NET analyses using the same methodology to examine presenting complaint have reported that the proportion of older children and adolescents admitted for non‐COVID‐19–related reasons may be higher than in adults [15, 16].

In addition to the availability of complete inpatient medical charts, determining presenting complaint on admission in COVID‐NET required trained staff as well as the development of algorithms and physician review to examine and classify the thousands of hospitalizations sampled in this analysis. Examining the chart fully for clinical characteristics, including for reason for admission written by the admitting provider in the chart and diagnoses written in the discharge summary, might provide a richer picture of the patient's course of hospitalization compared with other single‐source methods like data scraping or the use of administrative billing codes.

Using a multipart definition, including characteristics on admission, treatment course, and discharge diagnoses in a geographically diverse sample of hospitalizations found that no single characteristic examined in this study captured all hospitalizations defined as attributable to COVID‐19. Fewer than two thirds of hospitalized patients received COVID‐19–specific inpatient treatment or systemic steroids and fewer than half had a respiratory‐ or coagulopathy‐related discharge diagnosis or a principal diagnosis of COVID‐19. More than three quarters of likely COVID‐19–attributable hospitalizations and three quarters of hospitalizations with COVID‐19–specific treatment or outcomes had COVID‐19–related illness as the presenting complaint upon admission. While presenting complaint captured the vast majority COVID‐19–attributable hospitalizations on its own, about 15% of COVID‐19–attributable hospitalizations did not have COVID‐19–related illness as a presenting complaint. Hospital‐acquired infections may have contributed to this. In addition, while presenting complaint, as defined in this analysis, is an acceptable measure of hospitalization attributable to COVID‐19, determining presenting complaint upon admission can be difficult. Patients can present with a spectrum of signs and symptoms on admission; even among those who are asymptomatic on admission, COVID‐19 illness can evolve over the course of a hospitalization.

COVID‐19 can cause severe respiratory illnesses and a constellation of outcomes both directly attributable to SARS‐CoV‐2 infection, such as stroke, as well as exacerbations of underlying conditions. When assessing whether a hospitalization is attributable to COVID‐19, if this is not considered, public health surveillance systems are at risk of underestimating the frequency of hospitalizations attributable to COVID‐19. In addition, while this study classified some hospitalizations among SARS‐CoV‐2–positive patients as not COVID‐19 related, it is important to note that even if a patient's SARS‐CoV‐2–positive status was not considered to be directly related to hospital admission, COVID‐19–related illness could have influenced clinical decision‐making during hospitalization. Even without the presence of COVID‐19 illness, additional hospital resources are likely required to address SARS‐CoV‐2–positive patients [17], including isolation, exposure monitoring, and hospital staff exposure risk.

This analysis is subject to several limitations. First, SARS‐CoV‐2 testing is determined by provider and facility practices and is not systematic. Thus, some COVID‐19 cases might be missed due to not being tested but restricting testing to individuals with COVID‐19 symptoms could increase pre‐test probability of identifying positives; this could increase or decrease the proportion of COVID‐19–attributable hospitalizations. Furthermore, ICD‐10‐CM codes abstracted from clinical charts might be subject to coding error and are primarily intended for administrative and billing purposes, not surveillance, and could result in misclassification of COVID‐19–attributable hospitalizations. Additional misclassification might have occurred by including hospitalizations where the only criterion met for COVID‐19–attributable hospitalization was receipt of non‐steroidal antiviral treatment administered to hospitalized patients at high risk for progressing to severe COVID‐19 but with hospitalizations that would not otherwise meet our definition of COVID‐19–attributable. However, < 3% of COVID‐19–attributable hospitalizations in this analysis met this standard. Finally, while care was taken to use available data to accurately define the role of COVID‐19 in these hospitalizations, assessing this from medical charts is complex and misclassification could occur.

COVID‐19–attributable hospitalizations comprised nearly all SARS‐CoV‐2–positive hospitalizations occurring among adults during the pre‐Delta and Delta variant predominance periods (June 2020 to December 2021) but decreased in the early Omicron period (2022) among all ages, especially among patients aged 18–49 years. Though not statistically significant in most age groups, the subsequent modest increase in the proportion of COVID‐19–attributable hospitalizations in the later Omicron period might be linked to hospitals performing fewer COVID‐19 screenings. If the frequency of universal screening continues to decrease, fewer hospitalized asymptomatic persons or persons with presenting complaints likely unrelated to COVID‐19 who are positive for SARS‐CoV‐2 will be identified. Ongoing monitoring of testing practices and characteristics of patients hospitalized with SARS‐CoV‐2 will be critical to understanding the evolving burden of COVID‐19 disease.

Hospitalizations due to COVID‐19 continue to be a major public health problem, particularly among adults 65 years and older, a group that comprised two‐thirds of recent hospitalizations among SARS‐CoV‐2–positive persons. This study demonstrates that a modest proportion of hospitalizations among those who tested positive for SARS‐CoV‐2 could be attributable to other causes, particularly among younger adults. However, COVID‐19 likely plays a major role in most hospitalizations among those positive for SARS‐CoV‐2, particularly in the most severely impacted age groups that make up most COVID‐19 hospitalizations. Continued monitoring of changes and trends in both epidemiology and testing practices is critical. In addition, ongoing public health messaging about the potential severity of COVID‐19 is necessary, with an emphasis on vaccination and other preventative measures, as well as the importance of early outpatient treatment among those at high risk for severe disease.

## Author Contributions

**Christopher A. Taylor:** conceptualization, writing – original draft, visualization, methodology, project administration, investigation. **Michael Whitaker:** visualization, validation, formal analysis, conceptualization, data curation. **Monica E. Patton:** conceptualization, methodology. **Michael Melgar:** conceptualization, methodology. **Fiona P. Havers:** conceptualization, investigation, methodology, project administration, visualization, supervision. All authors also reviewed and edited the draft (writing – review and editing) and read and approved the final draft.

## Disclosure

The findings and conclusions in this report are those of the authors and do not necessarily represent the official position of the Centers for Disease Control and Prevention.

## Conflicts of Interest

The authors declare no conflicts of interest.

## Supporting information


**Figure S1.** Decision flowchart to determine COVID‐19–attributable hospitalization status among adults ages ≥ 18 years with laboratory‐confirmed SARS‐CoV‐2 test results—COVID‐19–Associated Hospitalization Surveillance Network (COVID‐NET). Presenting complaint upon admission was identified using information in the admission history and physical or face sheet. Respiratory‐related discharge diagnoses included acute respiratory distress syndrome (ARDS), acute respiratory failure, asthma exacerbation, bronchiolitis, bronchitis, chronic obstructive pulmonary disease (COPD) exacerbation, pneumonia, and sepsis. Coagulopathy‐related discharge diagnoses included acute myocardial infarction, deep vein thrombosis, disseminated intravascular coagulation (DIC), pulmonary embolism, stroke/cerebrovascular accident, and other thrombosis, embolism, and coagulopathy. ICD‐10‐CM refers to the International Classification of Diseases, Tenth Edition, Clinical Modification. Medications recommended for the therapeutic management of hospitalized adults with COVID‐19 included remdesivir, baricitinib, sarilumab, and tocilizumab.

**Figure S2.** Decision flowchart to determine presenting complaint on admission among adults ages ≥ 18 years with laboratory‐confirmed SARS‐CoV‐2 test results—COVID‐19–Associated Hospitalization Surveillance Network (COVID‐NET).

**Figure S3.** Percentage of hospitalizations likely attributable to COVID‐19 among adults ages ≥ 18 years with laboratory‐confirmed SARS‐CoV‐2, by age group and period of SARS‐CoV‐2 variant predominance—COVID‐19–Associated Hospitalization Surveillance Network (COVID‐NET), June 2020 to September 2023. Percentages are weighted to account for sampling with 95% confidence intervals indicated by error bars. Likely COVID‐19–attributable hospitalizations are defined as hospitalizations among laboratory‐confirmed SARS‐CoV‐2–positive patients with the test administered ≤ 14 days before or during admission with COVID‐19–related presenting complaint, treatment, or discharge diagnosis. Presenting complaint upon admission was identified using information in the admission history and physical or face sheet. Treatment included inpatient receipt of remdesivir, baricitinib, sarilumab, tocilizumab, or systemic steroids. Discharge diagnoses included respiratory‐related discharge diagnoses (acute respiratory distress syndrome [ARDS], acute respiratory failure, asthma exacerbation, bronchiolitis, bronchitis, chronic obstructive pulmonary disease [COPD] exacerbation, pneumonia, and sepsis) or coagulopathy‐related discharge diagnoses (acute myocardial infarction, deep vein thrombosis, disseminated intravascular coagulation [DIC], pulmonary embolism, stroke/cerebrovascular accident, and other thrombosis, embolism, and coagulopathy).

**Table S1.** Recoded categories for other specified presenting complaints as the result of clinician review and examples of defining terms used to identify cases within each category†—COVID‐NET.

## Data Availability

Data are not publicly available. Please contact corresponding author Christopher Taylor (iyq3@cdc.gov) with data‐related questions.

## Endnotes

1
*Signs and symptoms*. COVID‐19‐related signs and symptoms reported in the medical chart at admission included both respiratory and non‐respiratory signs and symptoms. Respiratory signs and symptoms included congestion, cough, hemoptysis, shortness of breath, sore throat, upper respiratory infection, influenza‐like illness, and wheezing; non‐respiratory‐related signs and symptoms included abdominal pain, altered mental status, anosmia, chest pain, conjunctivitis, diarrhea, dysgeusia, fatigue, fever/chills, headache, myalgia, nausea/vomiting, rash, and seizures.

2 See 45 C.F.R. part 46.102(I) [2], 21 C.F.R. part 56; 42 U.S.C. Sect. 241(d); 5 U.S.C. Sect. 552a; 44 U.S.C. Sect. 3501 et seq.
