# Supplementary material for: Trends in COVID‐19–Attributable Hospitalizations Among Adults With Laboratory‐Confirmed SARS‐CoV‐2—COVID‐NET, June 2020 to September 2023
Source: Influenza Other Respir Viruses. 2024 Nov 4;18(11):e70021. doi: 10.1111/irv.70021 (PMC11534501; doi:10.1111/irv.70021)
Supplement: Supplementary file 1 — Figure S1. Decision flowchart to determine COVID‐19–attributable hospitalization status among adults ages ≥ 18 years with laboratory‐confirmed SARS‐CoV‐2 test results—COVID‐19–Associated Hospitalization Surveillance Network (COVID‐NET). Presenting complaint upon admission was identified using information in the admission history and physical or face sheet. Respiratory‐related discharge diagnoses included acute respiratory distress syndrome (ARDS), acute respiratory failure, asthma exacerbation, bronchiolitis, bronchitis, chronic obstructive pulmonary disease (COPD) exacerbation, pneumonia, and sepsis. Coagulopathy‐related discharge diagnoses included acute myocardial infarction, deep vein thrombosis, disseminated intravascular coagulation (DIC), pulmonary embolism, stroke/cerebrovascular accident, and other thrombosis, embolism, and coagulopathy. ICD‐10‐CM refers to the International Classification of Diseases, Tenth Edition, Clinical Modification. Medications recommended for the therapeutic management of hospitalized adults with COVID‐19 included remdesivir, baricitinib, sarilumab, and tocilizumab. [file IRV-18-e70021-s003.pptx]

## Slide 1
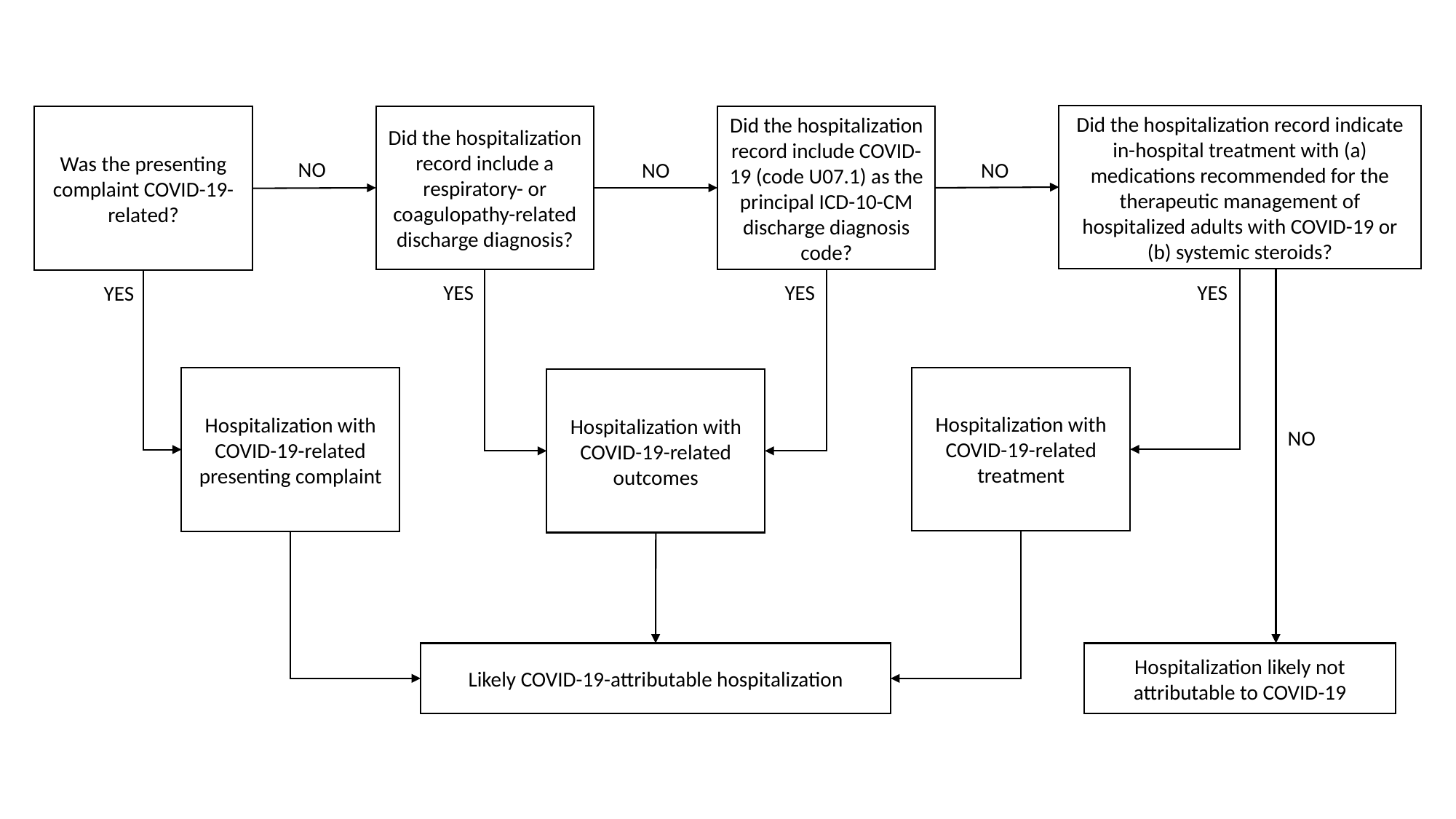

Did the hospitalization record indicate in-hospital treatment with (a) medications recommended for the therapeutic management of hospitalized adults with COVID-19 or (b) systemic steroids?
Did the hospitalization record include COVID-19 (code U07.1) as the principal ICD-10-CM discharge diagnosis code?
Did the hospitalization record include a respiratory- or coagulopathy-related discharge diagnosis?
Was the presenting complaint COVID-19-related?
NO
NO
NO
YES
YES
YES
YES
Hospitalization with COVID-19-related treatment
Hospitalization with COVID-19-related presenting complaint
Hospitalization with COVID-19-related outcomes
NO
Likely COVID-19-attributable hospitalization
Hospitalization likely not attributable to COVID-19
