# Supplementary material for: Trends in COVID‐19–Attributable Hospitalizations Among Adults With Laboratory‐Confirmed SARS‐CoV‐2—COVID‐NET, June 2020 to September 2023
Source: Influenza Other Respir Viruses. 2024 Nov 4;18(11):e70021. doi: 10.1111/irv.70021 (PMC11534501; doi:10.1111/irv.70021)
Supplement: Supplementary file 2 — Figure S2. Decision flowchart to determine presenting complaint on admission among adults ages ≥ 18 years with laboratory‐confirmed SARS‐CoV‐2 test results—COVID‐19–Associated Hospitalization Surveillance Network (COVID‐NET). [file IRV-18-e70021-s004.pptx]

## Slide 1
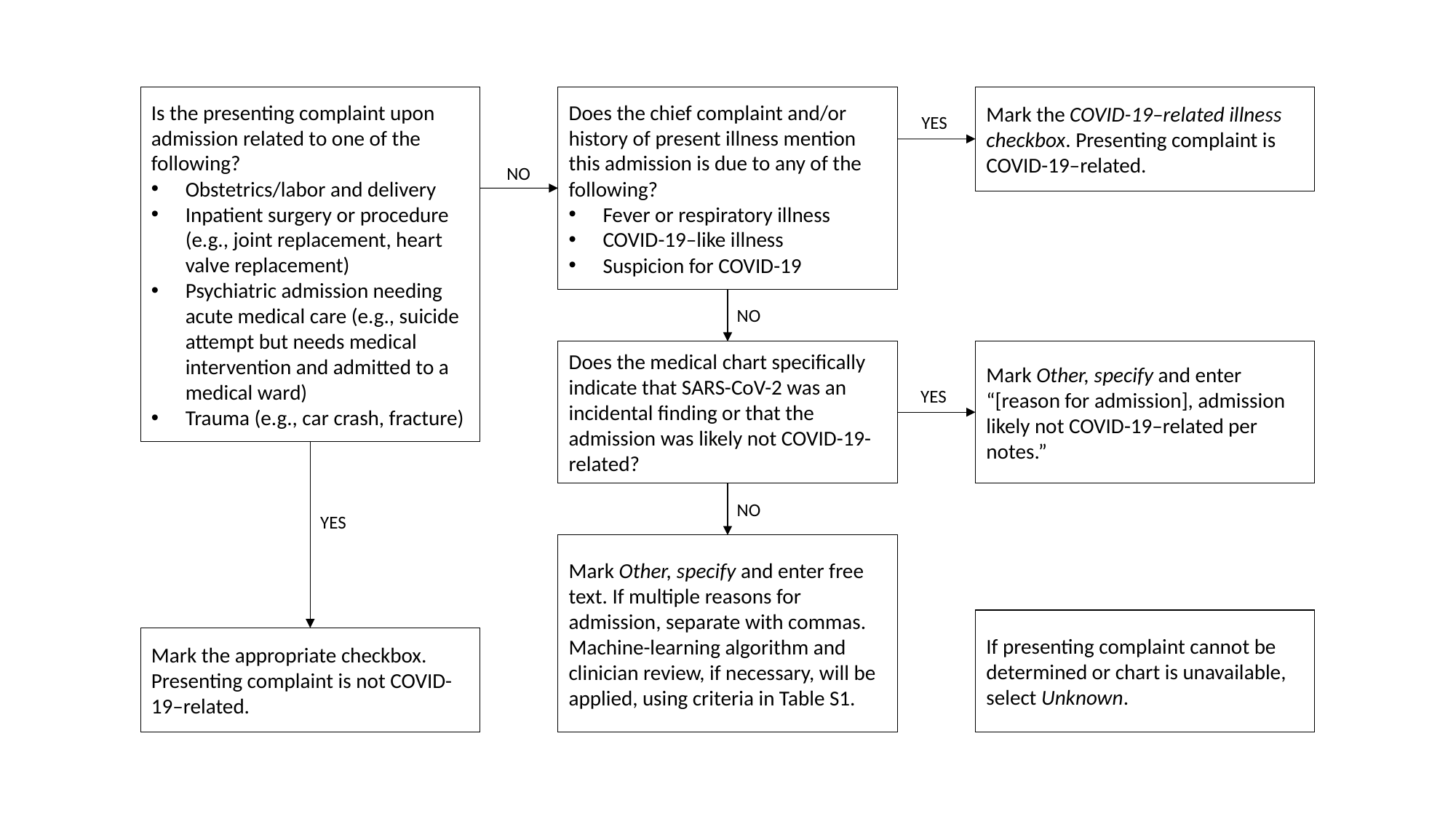

Is the presenting complaint upon admission related to one of the following?
Obstetrics/labor and delivery
Inpatient surgery or procedure (e.g., joint replacement, heart valve replacement)
Psychiatric admission needing acute medical care (e.g., suicide attempt but needs medical intervention and admitted to a medical ward)
Trauma (e.g., car crash, fracture)
Does the chief complaint and/or history of present illness mention this admission is due to any of the following?
Fever or respiratory illness
COVID-19–like illness
Suspicion for COVID-19
Mark the COVID-19–related illness checkbox. Presenting complaint is COVID-19–related.
YES
NO
NO
Mark Other, specify and enter “[reason for admission], admission likely not COVID-19–related per notes.”
Does the medical chart specifically indicate that SARS-CoV-2 was an incidental finding or that the admission was likely not COVID-19-related?
YES
NO
YES
Mark Other, specify and enter free text. If multiple reasons for admission, separate with commas.
Machine-learning algorithm and clinician review, if necessary, will be applied, using criteria in Table S1.
If presenting complaint cannot be determined or chart is unavailable, select Unknown.
Mark the appropriate checkbox. Presenting complaint is not COVID-19–related.
