# Supplementary figures and images for: Trends in COVID‐19–Attributable Hospitalizations Among Adults With Laboratory‐Confirmed SARS‐CoV‐2—COVID‐NET, June 2020 to September 2023
Source: Influenza Other Respir Viruses. 2024 Nov 4;18(11):e70021. doi: 10.1111/irv.70021 (PMC11534501; doi:10.1111/irv.70021)

## Slide 1
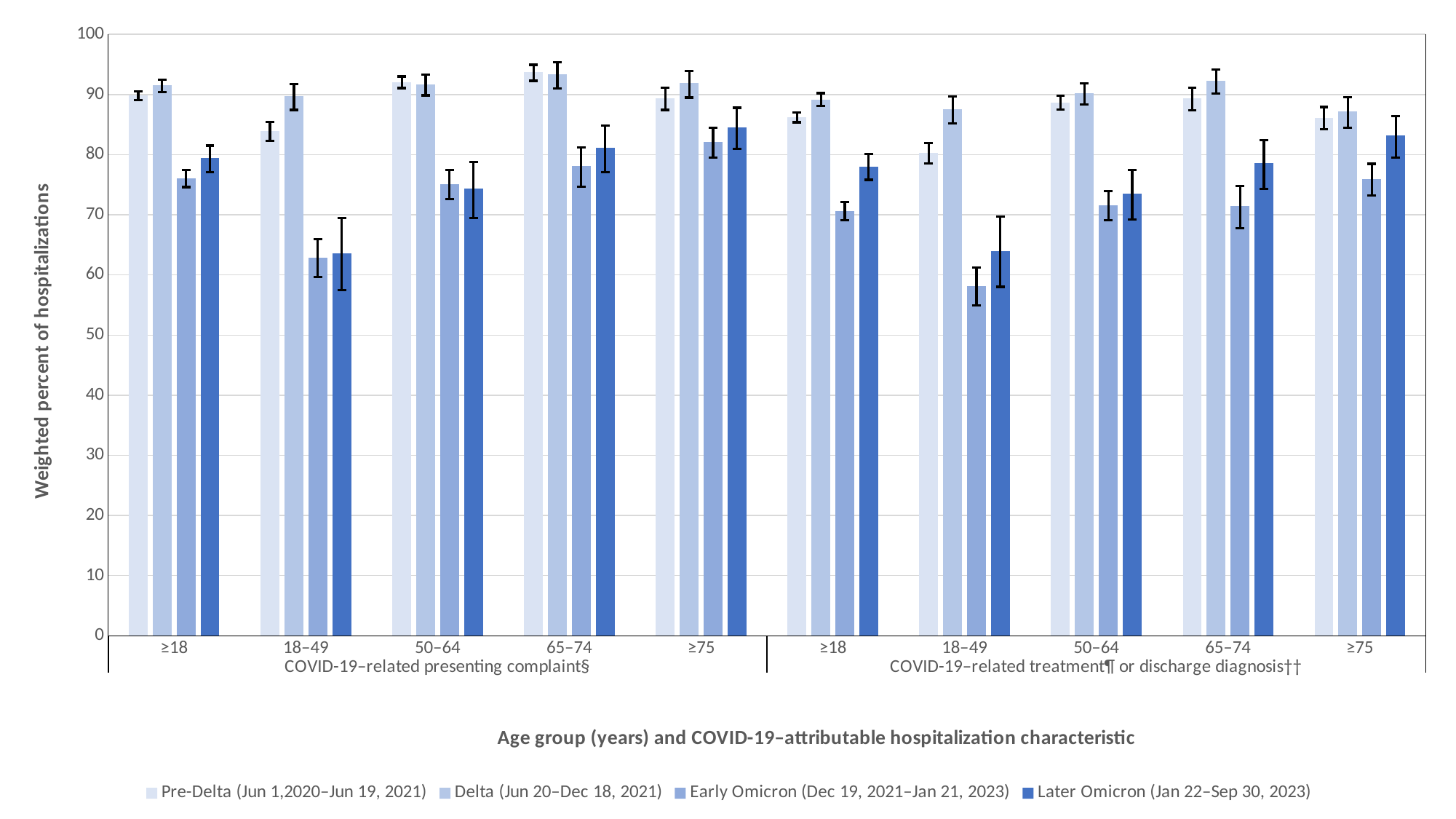

### Chart
| Category | Pre-Delta (Jun 1,2020–Jun 19, 2021) | Delta (Jun 20–Dec 18, 2021) | Early Omicron (Dec 19, 2021–Jan 21, 2023) | Later Omicron (Jan 22–Sep 30, 2023) |
|---|---|---|---|---|
| ≥18 | 89.8274999132326 | 91.4974557297841 | 76.0408761957225 | 79.3801181207023 |
| 18–49 | 83.8894775747381 | 89.723351800771 | 62.8298827559272 | 63.6000729511187 |
| 50–64 | 92.0729992581605 | 91.7243286415963 | 75.1033557178145 | 74.3041898056317 |
| 65–74 | 93.70865254721 | 93.3769214770281 | 78.0744267369564 | 81.1639497401043 |
| ≥75 | 89.4084045948496 | 91.8685319217005 | 82.1212798181094 | 84.5805269775452 |
| ≥18 | 86.1905025828197 | 89.1833392903288 | 70.6080761268614 | 78.0202941445678 |
| 18–49 | 80.2515452794937 | 87.5445033392408 | 58.0826718927699 | 63.9903227006968 |
| 50–64 | 88.6586072594159 | 90.1935901153079 | 71.5344786637358 | 73.516708346126 |
| 65–74 | 89.3359626721054 | 92.3212810331121 | 71.4089115165141 | 78.5428991218472 |
| ≥75 | 86.1404350692226 | 87.1783815412042 | 75.922111123701 | 83.1697514756039 |

Supplement: Supplementary file 3 — Figure S3. Percentage of hospitalizations likely attributable to COVID‐19 among adults ages ≥ 18 years with laboratory‐confirmed SARS‐CoV‐2, by age group and period of SARS‐CoV‐2 variant predominance—COVID‐19–Associated Hospitalization Surveillance Network (COVID‐NET), June 2020 to September 2023. Percentages are weighted to account for sampling with 95% confidence intervals indicated by error bars. Likely COVID‐19–attributable hospitalizations are defined as hospitalizations among laboratory‐confirmed SARS‐CoV‐2–positive patients with the test administered ≤ 14 days before or during admission with COVID‐19–related presenting complaint, treatment, or discharge diagnosis. Presenting complaint upon admission was identified using information in the admission history and physical or face sheet. Treatment included inpatient receipt of remdesivir, baricitinib, sarilumab, tocilizumab, or systemic steroids. Discharge diagnoses included respiratory‐related discharge diagnoses (acute respiratory distress syndrome [ARDS], acute respiratory failure, asthma exacerbation, bronchiolitis, bronchitis, chronic obstructive pulmonary disease [COPD] exacerbation, pneumonia, and sepsis) or coagulopathy‐related discharge diagnoses (acute myocardial infarction, deep vein thrombosis, disseminated intravascular coagulation [DIC], pulmonary embolism, stroke/cerebrovascular accident, and other thrombosis, embolism, and coagulopathy). [file IRV-18-e70021-s001.pptx]
