# Supplementary material for: Trends in COVID‐19–Attributable Hospitalizations Among Adults With Laboratory‐Confirmed SARS‐CoV‐2—COVID‐NET, June 2020 to September 2023
Source: Influenza Other Respir Viruses. 2024 Nov 4;18(11):e70021. doi: 10.1111/irv.70021 (PMC11534501; doi:10.1111/irv.70021)
Supplement: Supplementary file 4 — Table S1. Recoded categories for other specified presenting complaints as the result of clinician review and examples of defining terms used to identify cases within each category†—COVID‐NET. [file IRV-18-e70021-s002.docx]

**Table S1.** Recoded categories for other specified presenting complaints as the result of clinician review and examples of defining terms used to identify cases within each category† — COVID-NET

| **Other specified presenting complaint** | **Recoded category** | **Examples of defining terms‡** |
| --- | --- | --- |
| Abdominal/epigastric pain not attributed to a specific cause | COVID-19 | Vomiting, bloody stool, testicular pain, ileus |
| Acute diabetic issues | COVID-19 | Diabetic ketoacidosis (DKA), hyperglycemia, hypoglycemia |
| Alcohol or drug withdrawal, detox, or overdose | Psychiatric |  |
| Anemia, any type | Other |  |
| Animal bite | Trauma |  |
| Appendectomy | Planned inpatient procedure or surgery |  |
| Appendicitis | Other |  |
| Bacteremia, positive blood cultures, need for antibiotics | COVID-19 |  |
| Biliary-related | Other | Cholecystitis, choledocholithiasis, cholelithiasis, bile duct stone |
| Cancer | Other | Mass, chemotherapy, leukemia |
| Cardiac-related | COVID-19 | Cardiac arrest, cardiac shock, chest pain, heart failure, hypertensive or hypotensive emergency, irregular heartbeat, myocardial infarction, non–ST–elevation myocardial infarction (NSTEMI), palpitations |
| Cellulitis, wound, ulcer, abscess | Other |  |
| Cirrhosis | Other |  |
| Dialysis, line infection, IV placement | Other |  |
| Diarrhea | COVID-19 |  |
| Dizziness | COVID-19 |  |
| Eclampsia/preeclampsia | Obstetric/gynecological/labor and delivery |  |
| Failure to thrive | COVID-19 | Inability or unable to eat, difficulty eating, malnutrition |
| Fall | COVID-19 | “Found down,” syncope, syncopal, unresponsive; includes those that mention alcohol or drug use |
| Flank pain | COVID-19 |  |
| Fracture | Trauma |  |
| Gastrointestinal–specific issues without mention of abdominal pain | Other | Constipation, colitis, bowel obstruction, rectal pain/bleeding, melena, bloody/tarry stools, gastrointestinal bleed, vomiting blood |
| Genitourinary (pyelonephritis, UTI, hematuria, painful urination, urinary problem, urinary retention, kidney stone, nephrotic syndrome, end stage renal disease, vaginal bleeding) | Other |  |
| Gestational hypertension | Obstetric/gynecological/labor and delivery |  |
| Headache | COVID-19 |  |
| Immobility | COVID-19 |  |
| Jaundice | COVID-19 |  |
| Kidney failure | COVID-19 | Bilateral leg, feet, or ankle swelling or edema |
| Lethargy | COVID-19 |  |
| Mental status changes | COVID-19 | Confusion, dementia, hallucinations, encephalopathy; includes those that mention alcohol or drug use |
| Motor vehicle accident | Trauma |  |
| “Multiple” | COVID-19 |  |
| Nausea or vomiting | COVID-19 |  |
| Neck/parotid swelling | COVID-19 |  |
| Neuropathy, fatigue, weakness (including ataxia, | COVID-19 |  |
| Numbness | COVID-19 |  |
| Pain, including back, hand, feet, arm, leg, neck/shoulder, eye, or pelvic pain | Other |  |
| Pancreatitis | COVID-19 |  |
| Seizure | COVID-19 |  |
| Sickle cell crisis | COVID-19 |  |
| Stroke/cerebrovascular accident | COVID-19 |  |
| Subdural hematoma | Trauma |  |
| Suicidal ideation | Psychiatric |  |
| Surgery complications | Other |  |
| Unsteady gait | COVID-19 |  |

† A natural language processing machine learning algorithm was developed to categorize presenting complaints initially categorized as “other” by examining the specified free text to determine if it should be recategorized as one of the specific categories, including COVID-19-related illness, or left as “other.” To develop the algorithm, three clinicians not involved in the treatment of the patient and independent of the medical record abstraction reviewed the free-text field of complaints classified as “other” using agreed upon rules to guide categorization. For classifications not in agreement across all reviewers, adjudication was done to reach consensus. Successive applications of the machine learning algorithm to new data were reviewed and any previously uncategorized complaints are categorized and incorporated into the algorithm using the specified process.

‡ The examples of defining terms listed are not intended to be a complete list of terms. Similar, related terms, in combination with clinical judgment from the review panel, might have been utilized to categorize cases where the presenting complaint was initially coded as “other.”
